# The prevalence of canine dirofilariasis in China: a systematic review and meta-analysis

**DOI:** 10.1186/s13071-023-05770-9

**Published:** 2023-06-20

**Authors:** Zhu Ying, Archana Upadhyay, Jinhua Wang, Qian Han, Qun Liu

**Affiliations:** 1 One Health Institute, Hainan University, Haikou, 570228 Hainan China; 2 National Animal Protozoa Laboratory, College of Veterinary Medicine, China Agricultural University, Beijing, 100083 China; 3 Laboratory of Tropical Veterinary Medicine and Vector Biology, School of Life Sciences, Hainan University, Haikou, 570228 Hainan China; 4 School of Animal Science and Technology, Hainan University, Haikou, 570228 Hainan China

**Keywords:** Dirofilariasis, Canine, China, Meta-analysis, Prevalence

## Abstract

**Background:**

Dirofilariasis, the disease caused by *Dirofilaria* spp., and in particular by *Dirofilaria immitis* and *Dirofilaria repens* in canines, occurs frequently in canids and felids, and occasionally in humans, in temperate, sub-tropical and tropical regions globally. Although highly effective, safe and convenient preventive medicines have been available for the treatment of dirofilariasis for the past three decades, the disease remains a major veterinary and public health concern in endemic areas. The insect vectors, host-parasite relationships and interactions of *Dirofilaria* spp. have received little attention in China, and there is very little information in English regarding the prevalence of dirofilariasis in animals and humans in the country. The aim of this systematic review and meta-analysis is to evaluate the status of canine dirofilariasis in China based on the available literature in English and in Chinese.

**Methods:**

We systematically searched five databases for epidemiologic studies on the prevalence of canine dirofilariasis in China and finally selected 42 studies eligible for inclusion in the systematic review and meta-analysis. The meta-analysis was performed using the random effects model in the meta package in R v4.2.1.

**Results:**

The random effects model gave a pooled and weighted prevalence of *Dirofilaria* infection among dogs in China in the past 100 years of 13.8% (2896/51,313, 95% confidence interval 8.2–20.4%) with a high level of heterogeneity (*I*^*2*^ =  99.5%).

**Conclusions:**

Our analyses indicated that the prevalence of canine dirofilariasis in China has gradually declined, but that the range of *Dirofilaria* spp. has expanded. Older and outdoor dogs presented a higher rate of positive infection. The findings indicated that more attention should be paid to host factors for the effective control and management of this disease.

**Graphical Abstract:**

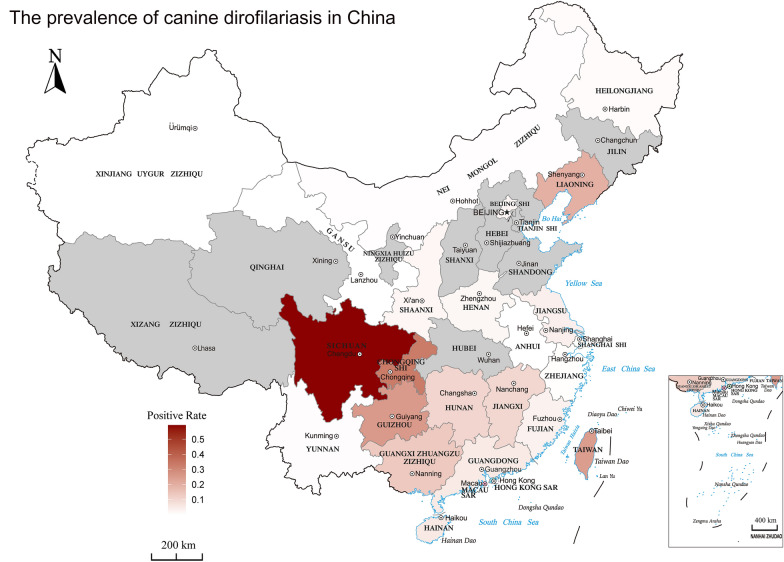

**Supplementary Information:**

The online version contains supplementary material available at 10.1186/s13071-023-05770-9.

## Background

Dirofilariasis is mainly caused by the parasitic nematodes *Dirofilaria immitis* and *Dirofilaria repens*, which are transmitted by different species of culicid mosquitoes (*Culex* spp., *Aedes* spp., *Anopheles* spp.), the vectors that allow these nematodes to complete their life cycles [20]. *Dirofilaria immitis* and *D. repens* infect canines, felines and other animals, including humans, mostly in temperate, sub-tropical and tropical areas worldwide. In dogs, the adult *D. immitis* worms are located in the pulmonary arteries and right ventricle, and are capable of causing life-threatening cardiopulmonary disease [12]. Adult *D. repens* worms are found in subcutaneous tissues and subconjunctival, pulmonary and peri-muscular connective fascia in dogs; in most cases infection is asymptomatic, although some infections give rise to subcutaneous nodules and allergic dermatitis [1, 16]. In the majority of cases of *D. repens* infections in humans, individuals present with nodules in the tissues [13, 54]. Both *D. immitis* and *D. repens* are of worldwide concern as they are considered to be agents of human dirofilariasis [10, 25, 47]. Most epidemiological studies on dirofilariasis in dogs and cats, and also on human cases of the disease, have been carried out in the USA, Japan, Europe, Russia and Australia. The usual definitive hosts of *Dirofilaria* spp. are primarily wild and domestic canids, and the prevalence of canine dirofilariasis has been increasing in the past 10 years in countries that were previously considered non-endemic. Various factors, such as environmental and climatic changes, an increase in mosquito populations, and more human and animal movement, have favored the recent increase in infections in regions where the parasites were previously endemic, and also their spread to geographic areas which were previously free of canine infections [34].

Canine dirofilariasis is endemic in China. Faust first reported heartworm infections in dogs in China in 1921 [11]. A few researchers continued the study of heartworm, but mainly focused on its epidemiology. Data on the prevalence and spread of canine heartworm infections in China have been reported in the Chinese literature in several studies, but reports on *Dirofilaria* infection in China are very scarce in the international literature. To the best of our knowledge, there are only a few epidemiological surveys on *D. immitis* in China, while for *D. repens*, there are only a few case reports of human infections. The main objectives of this systematic review were to summarize the available data on the epidemiology of canine *Dirofilaria* infections in China using a meta-analysis and to further examine the possible causes of documented changes in the occurrence and distribution of the disease.

## Methods

The preferred reporting items for systematic reviews and meta-analyses [37] were strictly adhered to for the systematic review and meta-analysis presented here.

### Literature search strategy

All the articles were retrieved from major databases that predominantly include publications in the English language (PubMed and Web of Science) or in the Chinese language (China National Knowledge Infrastructure, database of Chinses Science and Technology Periodicals, and Wan Fang database) using medical subject heading terms and corresponding keywords such as “prevalence,” “dirofilariasis,” “*Dirofilaria immitis*,” “*Dirofilaria repens,*” and “China”. The keywords “parasite” and “worm” were also searched for, and the references of the published articles were also checked for any additional useful information.

### Selection criteria, quality assessment and data extraction

All cross-sectional studies based on the prevalence of canine dirofilariasis in China published before December 2022 with full-text accessibility were separately evaluated by two reviewers, and any contradictions with respect to the selection process were resolved by a third reviewer; thus there were three reviewers in all. The extracted data included the details of the authors, sampling locations, sampling times, sample sizes, prevalence, diagnostic tests and risk factors.

A scoring approach was used to assessed the quality of the papers. One point was given when the study met the following criteria and zero points were given if it did not: (1) complete information of the study, (2) a detailed materials and methods section, (3) random sampling, (4) sampling period within a given time span, (5) data analysis included, (6) missing data discussed, and (7) an analysis of risk factors [69]. The highest possible score was seven, and articles awarded five points or more were considered to be of high quality.

### Statistical analysis

Double-arcsine transformation was used to convert prevalence to meet the conditions of a normal distribution, and forest plots were used for data visualization. Cochran’s *Q* and the *I*^*2*^ index were used to assess data heterogeneity among studies. As there was high heterogeneity, with *I*^*2*^-values above 75%, a random effects model was used to perform the meta-analysis. To assess the possible causes of heterogeneity, subgroup analysis was performed according to region, province, sampling time, quality score and latitude. A funnel plot and a trim-and-fill analysis were performed to check for publication bias based on Egger’s test [9]. The association between *Dirofilaria* infection and possible risk factors was estimated via odds ratio (OR) as follows: age (< 3 years compared to > 3 years), sex (female compared to male), management practice (dogs kept indoors compared to outdoors) and breed (purebred compared to crossbreed). A *P*-value < 0.05 was considered statistically significant. The meta-analysis was performed using R v4.2.1 and the meta package.

## Results

### Literature search and eligible studies

**Fig. 1:**
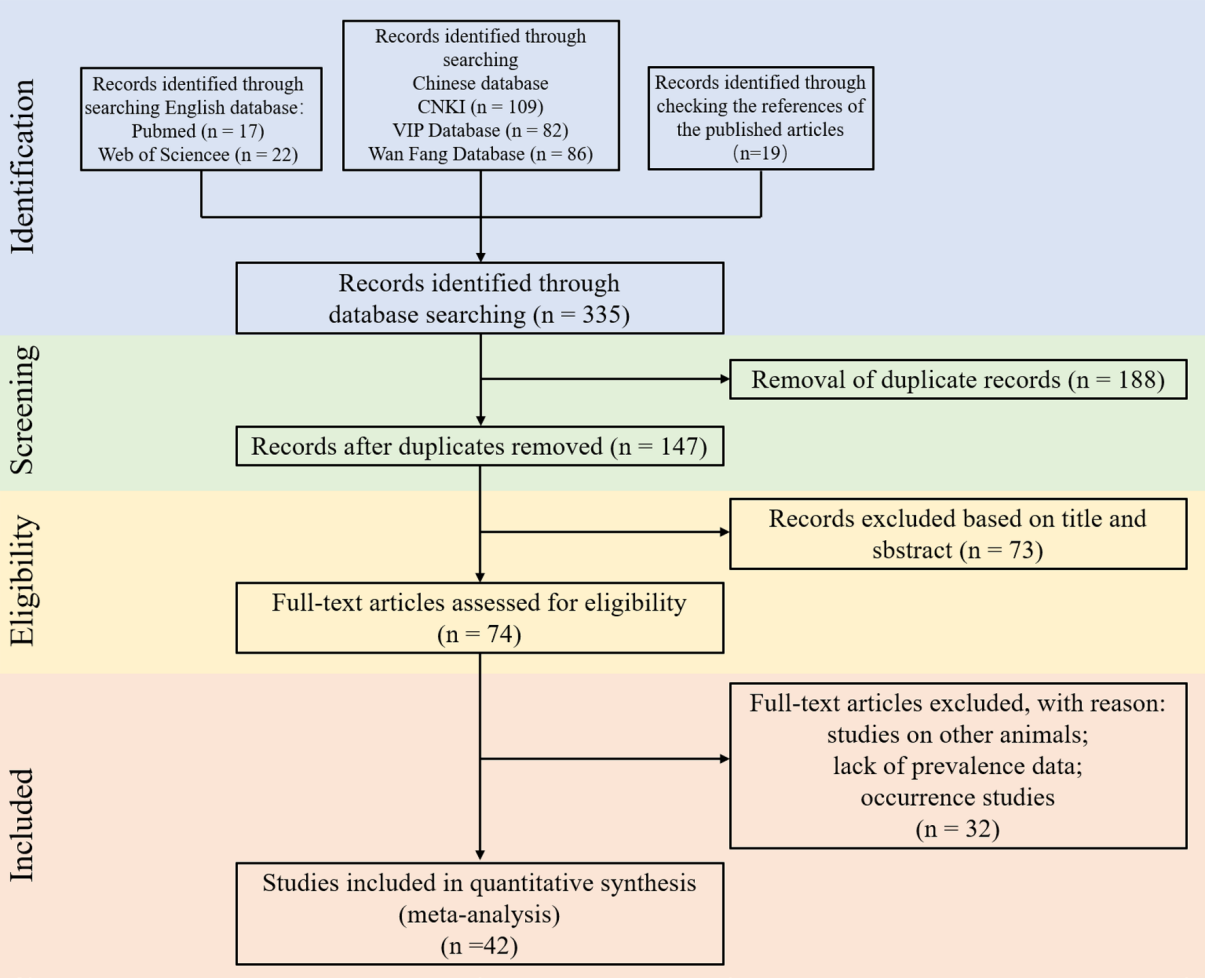
Preferred reporting items for systematic reviews and meta-analyses flow diagram of the process used here to determine the prevalence of canine dirofilariasis in China.* CNKI* China National Knowledge Infrastructure database,* VIP* database of Chinese Science and Technology Periodicals

A total of 335 articles were retrieved using the literature search strategy, of which only 42 eligible studies were used in the meta-analysis. These comprised 16 articles in English and 26 articles in Chinese (Fig. 1; Additional file 2: Table S1; Additional file 3: Table S2) [33, 43, 50, 51, 62, 66, 76–111]. According to the quality assessment, 26 were allocated five or more points, and were thus considered of high quality, and 16 were allocated five or fewer points, and were thus considered of low quality (Additional file 2: Table S1).

### Meta-analysis and prevalence assessment

**Fig. 2:**
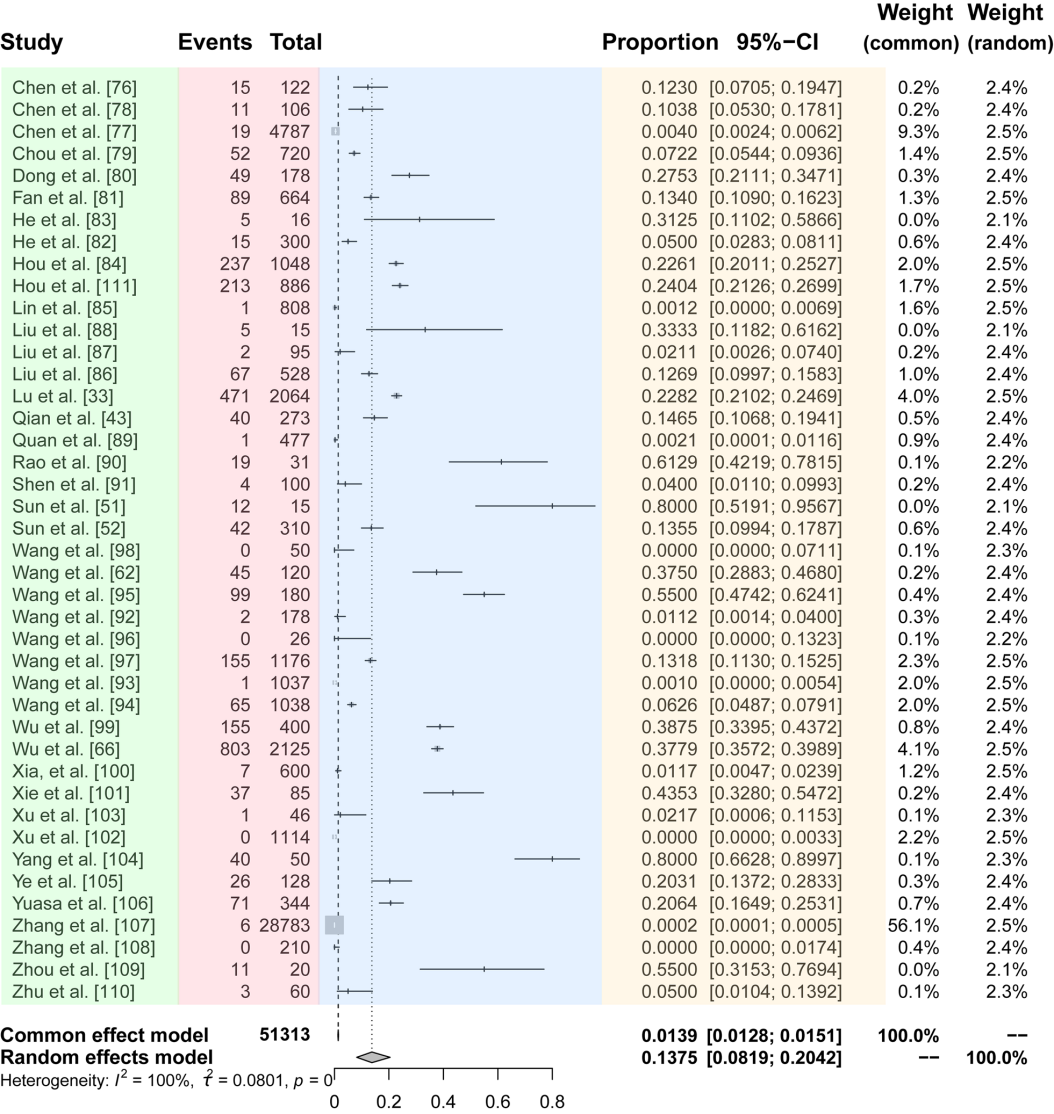
Forest plot of the prevalence of canine dirofilariasis in China

The random effect model gave a pooled and weighted prevalence of *Dirofilaria* infection among dogs in China of 13.8% [95% confidence interval (CI) 8.2–20.4%] with high heterogeneity *Q* =  9051.95, *P* = 0, *I*^*2*^ =  99.5%). A total of 51,313 dogs were investigated for dirofilariasis, of which 2896 were positive for *Dirofilaria* infection (Fig. 2). Both the funnel plots and the trim-and-fill analysis indicated a publication bias in the meta-analysis (Additional file 1: Figure S1A, C), with Egger’s test showing a significant publication bias (Egger’s bias = 10.8474, *P* = 0.0001; Additional file 1: Figure S1B). However, no individual study significantly affected the pooled prevalence according to the sensitivity analysis (Additional file 1: Figure S1D).

### Meta-analysis

**Fig. 3:**
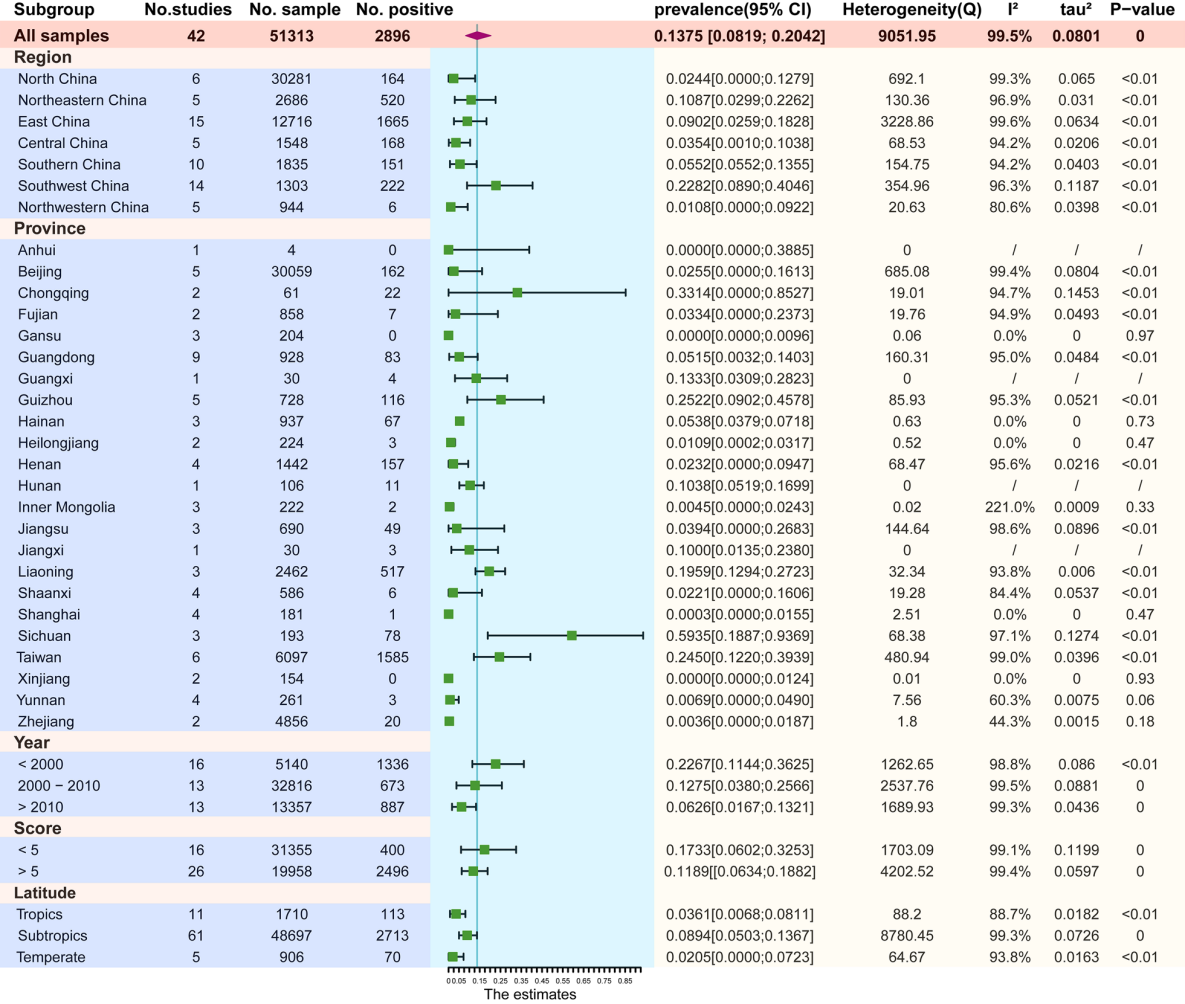
Forest plot of the subgroup analysis for *Dirofilaria* infection according to region, province, sampling time, quality score and latitude

There were difference in prevalence between different region subgroups in China (*P* = 0.0703). The lowest prevalence was in northwestern China (6/944, 1.1%, 95% CI 0.0–9.2%) and the highest in southwestern China (222/1303, 22.8%, 95% CI 8.9–40.5%; Fig. 3).

**Fig. 4:**
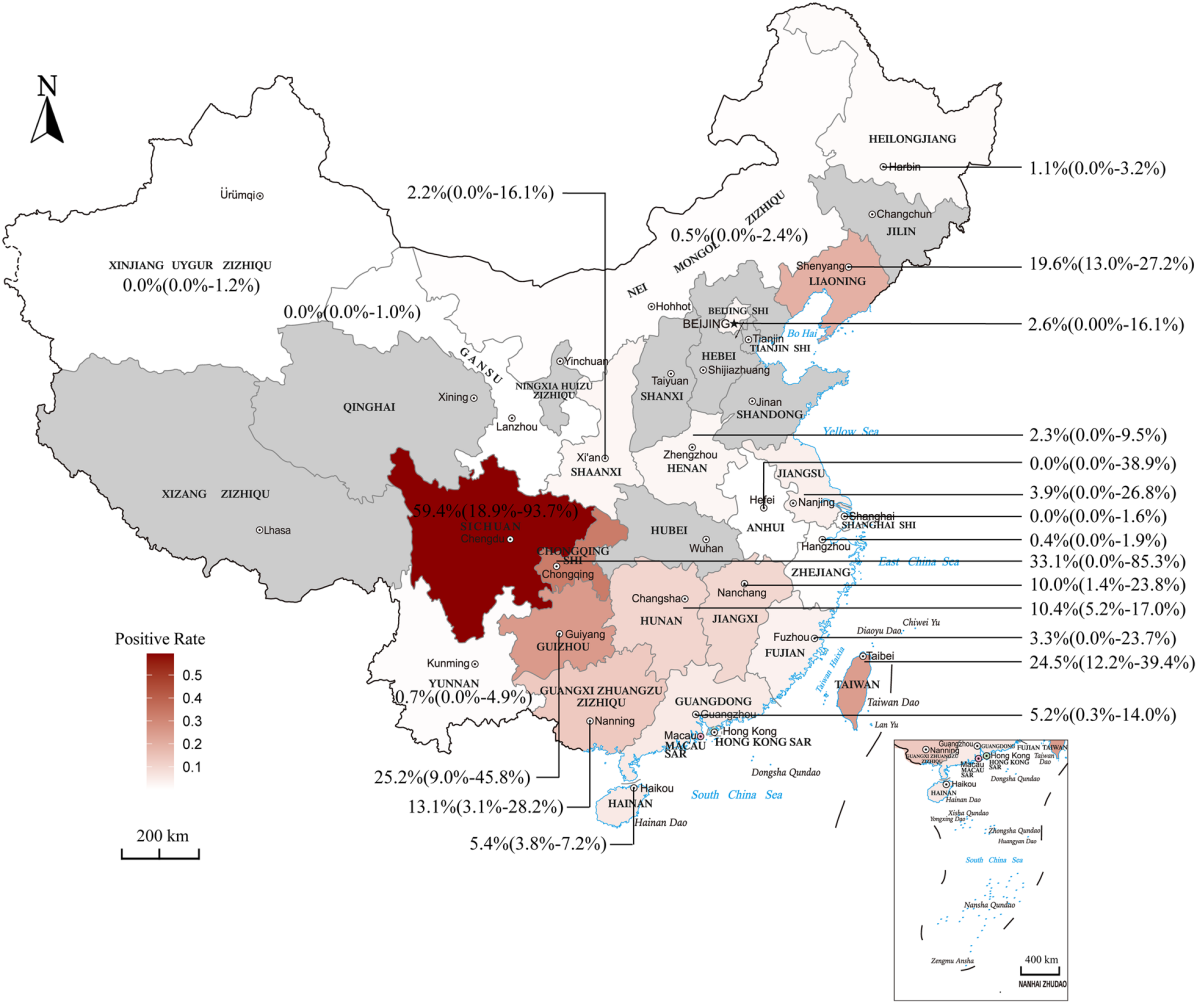
Map showing the prevalence of canine dirofilariasis in China

Significant differences were observed in prevalences between different provinces or cities in China (*P* < 0.0001), and these had a wide range (0.0–59.4%). The prevalence was lowest in Anhui (0/4, 0.0%, 95% CI 0.0–38.9%), followed by Gansu (0/204, 0.0%, 95% CI 0.0–1.0%) and then Xinjiang (0/154, 0.0%, 95% CI 0.0–1.2%), and highest in Sichuan (78/193, 59.4%, 95% CI 18.9−93.7%; Figs. 3, 4).

The rate of positive heartworm infection in dogs gradually decreased over time (*P* = 0.0639; Fig. 3). Prevalence was 22.7% before 2000 (1336/5140, 95% CI 11.4–36.2%), and 12.8% between 2000 and 2010 (673/32,816, 95% CI 3.8–25.7%). After 2010, the infection rate had reduced to 6.3% (887/13,357, 95% CI 1.7–13.2%).

The quality score was not an important factor with respect to prevalence (*P* = 0.4211; Fig. 3). The pooled rate of low-quality studies was 17.3% (400/31,355, 95%CI 6.0–32.5%), whereas the rate of high-quality studies was 11.9% (2496/19,958, 95% CI 6.3–18.8%).

**Fig. 5:**
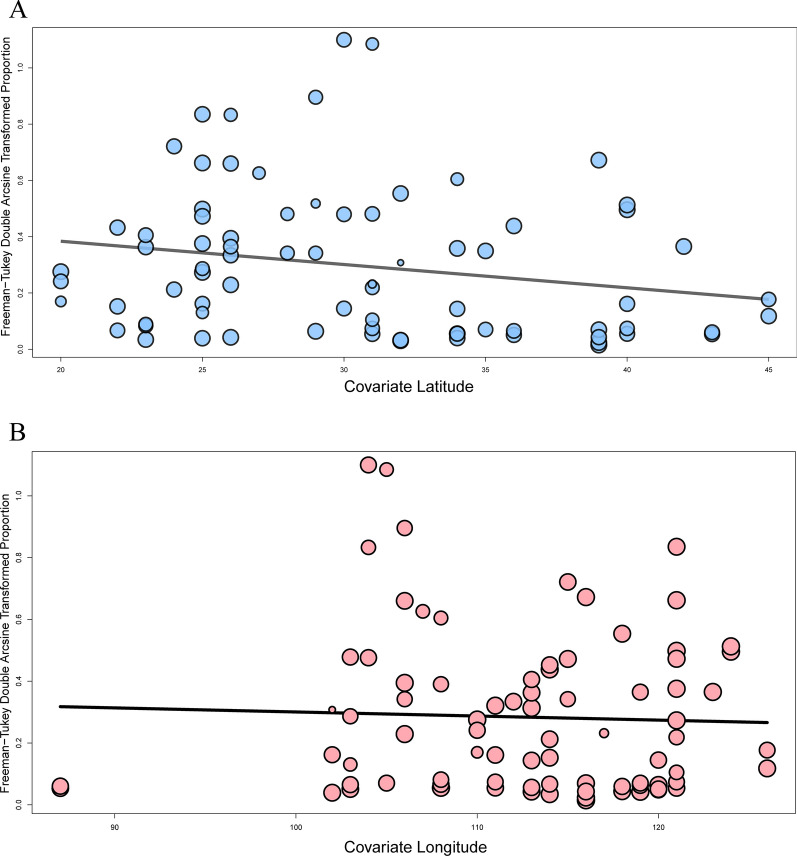
Meta-regression plot of latitude (**a**) and longitude (**b**) of the studied regions against the prevalence of canine dirofilariasis [percentage of seropositivity (*y*-axis)]. Circles represent individual studies. The continuous line is the regression line

The prevalence of dirofilariasis differed significantly between different thermal belts (*P* = 0.035; Fig. 3). Compared to the tropical (113/1710, 3.6%, 95% CI 0.7–8.1%) and temperate zones (70/906, 2.1%, 95% CI 0.0–7.2%), the subtropical zone (2713/48,697, 9.0%, 95% CI 5.0–13.7%) had the highest infection rate. According to the meta-regression, there was a relationship between geographic latitude and *Dirofilaria* infection (*P* = 0.0601; Fig. 5a). However, there was no effect of longitude on infection rate (*P* = 0.7225; Fig. 5b).

The subgroup analysis showed that region, province, sampling year and latitude may have been major sources of data heterogeneity.

### Risk factors

**Fig. 6:**
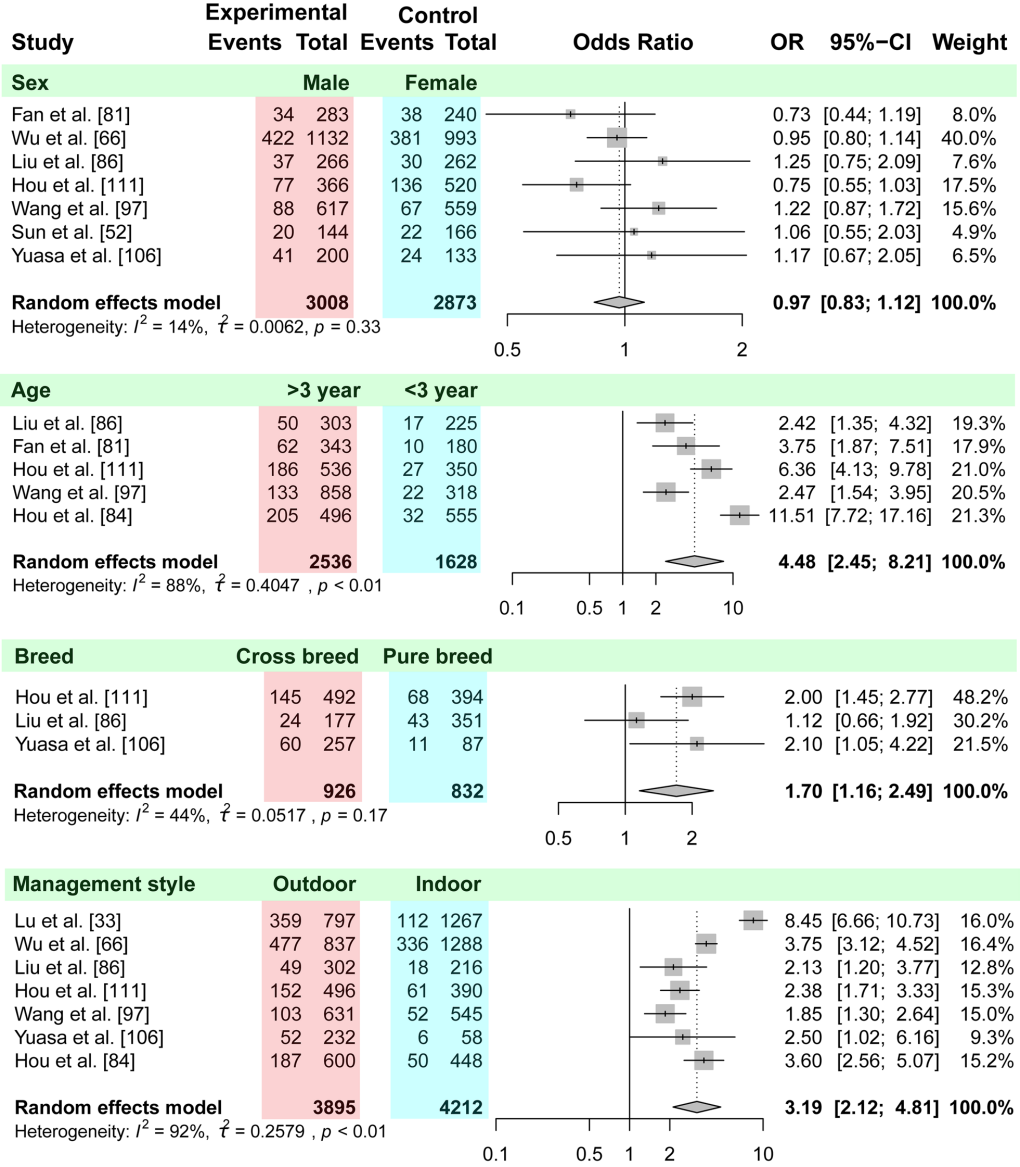
Forest plot of the association between heartworm infection and risk factors. Experimental group (*red*) compared to control group (*blue*): sex (female compared to male), age (< 3 years compared to > 3 years), breed (pure breed compared to crossbreed) and management practice (indoor compared to outdoor)

Very few studies on risk factors related to *Dirofilaria* infection have been reported for China. The studies were mainly focused on sex, age, breed and feeding mode. The results from the meta-analysis using data from five studies confirmed that dogs older than 3 years were more susceptible to heartworm infection than dogs younger than 3 years (OR 4.48, 95% CI 2.45–8.21, *P* < 0.01; Fig. 6). Dogs kept outdoors were three times more susceptible to the infection than those kept indoors (OR 3.19, 95% CI 2.12–4.81, *P* < 0.01). Data on the association between dirofilariasis and sex were extracted from seven studies; the results of the meta-analysis did not show any significant association between them (OR 0.97, 95% CI 0.83–1.12, *P* = 0.33). No difference was indicated between purebred and crossbreed dogs (OR 1.70; 95% CI 1.16–2.49; *P* = 0.17).

## Discussion

Despite the application of many preventive and precautionary measures, dirofilariasis continues to cause severe and fatal disease outcomes in dogs and other animals, and also affects humans due to its zoonotic nature. The infective third-stage larvae (L3) are transmitted to vertebrate hosts through the bites of infected mosquitoes, and *Dirofilaria* can spread into geographical areas where they have not been reported previously via these vectors [15]. As dogs are the normal definitive hosts of *Dirofilaria* spp., the evaluation of the infection rates of canines in a geographical area can help researchers understand the transmission of dirofilariasis and its intensity in that area.

In the Americas, several *Dirofilaria* species have been reported from domestic and wild mammals. *Dirofilaria immitis* is the most important causative agent of canine dirofilariasis, and is found in most parts of the Americas, except for Chile, French Guiana and Uruguay [7]. Its prevalence ranges from 1 to 12% in the USA, and is as high as 42% in the cities on the Gulf Coast of Mexico, 45% in Brazil, and 74% in Argentina [26–28, 59, 60]. In Europe, the highest prevalences have been reported in countries in the south of the continent, such as Italy (6.1%)[42], Spain (19.4%) [38] and Portugal (2.1%), which have historically been considered to be endemic/hyperendemic for dirofilariasis. An expansion of cardiopulmonary dirofilariasis in dogs toward central and northern Europe has been observed [39]. In Africa, both *D. immitis* and *D. repens* have been reported from Tunisia [46], Algeria [55] and Tanzania [40], with prevalences ranging from 1.4% to 14.5%. The presence of dirofilariasis in domestic dogs and dingoes has been well documented in Australia, where the prevalence of *D. immitis* was 15% in domestic dogs and 72.7% in dingoes in 2001 [49].

It has been more than 100 years since Faust first discovered *Dirofilaria* infections in dogs in China [11]. Although many cases of canine dirofilariasis have been reported for China, few of them were investigated epidemiologically, with reports limited to only certain regions or provinces. Thus reports and data on the prevalence of *Dirofilaria* infections among dogs in China are lacking. What is more, in China, epidemiological investigations have only been conducted on *D. immitis*, not on *D. repens*. To our knowledge, the meta-analysis present here is the first to summarize the pooled infection rate of canine dirofilariasis in China over the past 100 years. A total of 42 eligible studies were included in this meta-analysis, through which we could retrieve data from 51,313 dogs. The pooled rate in China was found to be 13.8% (95% CI 8.2-20.4%). The prevalence, which was mainly based on data on *D. immitis*, was higher than the pooled rate of *Dirofilaria* infection among dogs in China as reported by Anvari [2], which gave a weighted prevalence of 8.8% (95% CI 2.1-19.6%). We included 26 Chinese articles in the present review, including a paper published in 1956, in which all the relevant data may have reflected the actual situation of heartworm infection in China at the time when the papers were published.

Among the seven regions of China, the prevalence of *D. immitis* was highest in southwest China (222/1303, 22.8%, 95% CI 8.9–40.5%), which comprises Chongqing, Sichuan, Guizhou, Yunnan and Tibet province/autonomous region. Sichuan province was found to be endemic for canine heartworm due to its climatic conditions, as they are particularly suitable for the mosquito vectors which transmit the infective L3. Moreover, in this review, Sichuan was found to have the highest infection rate of the seven regions. A prevalence of *D. immitis* as high as 80% was reported for Sichuan province, in Cangxi county [51]. The prevalence of *D. immitis* in Sichuan province has fallen to 20.3% since 2000, and the highest prevalence was found in Mianyang (21.9%) [68]. The Patriotic Health Movement initiated by the Chinese government to eliminate mosquitoes is the main reason for the decline of *Dirofilaria* infections in Sichuan province. Chongqing municipality, one of the four municipalities of China, is located east of Sichuan province, which is endemic for heartworm. Rao et al. [45] reported that 19 out of 31 (i.e. an infection rate of 61.3%) stray dogs sampled between 1992 and 1993 in Bishan county, Chongqing, had heartworm microfilariae as determined by microscopy. Prior to the start of the twenty-first century, dirofilariasis was prevalent throughout Guizhou province, which is located southeast of Sichuan province, due to its warm, wet climate. A survey conducted from 1993 to 1994 in Yuqing County, Guizhou province, reported an infection rate of canine heartworm of 33.3% [32]. In Guizhou province, necropsy showed that 45 out of 120 stray dogs harbored adult *D. immitis* worms in the right ventricle and pulmonary artery, which represented an infection rate of 37.5% [62]. A survey undertaken in 1996 in Baiyun, Yuqin, Xifeng, Leishan, Jianhe, Xingyi, Bijie, Weining and Liuzhi, which are cities/counties of Guizhou province, reported a heartworm infection rate of 14.7% [43]. In 2012, the prevalence of heartworm was 6.7% in Yunnan, where previously dirofilariasis had not been reported at all [52].

Of the 34 provinces/autonomous regions/municipalities of China, only 23 possess epidemiological data on heartworm disease, and a few, like Tibet, Qinghai, Ningxia, Shanxi, Hubei, Hebei, Shandong, and Jilin, still have no relevant data. However, a few unpublished clinical cases of *D. immitis* infection in dogs have been reported in these provinces/autonomous regions. There are only a few reports in the Chinese literature of clinical cases in other cities of Jilin province, such as Longjing city, Changchun city and Yushu city [35, 71]. From 2000 to 2019, six reports on heartworm infection in dogs in Shandong were published in Chinese, and comprised one case each of an infected dog in 2000 [8], 2006 [18], 2010 [23], and 2019 [30], three cases of bulldogs infected with heartworm in 2003 [73], and a number of cases of canine dirofilariasis in 2004 [65]. Furthermore, in 2007, two Manchurian tigers were found to be infected with heartworm [63]. Canine heartworm cases have been reported in Hebei province, northern China [72]. Although the available data show that the rates in Anhui, Gansu and Xinjiang were 0.0%, the CIs differed due to different sample sizes.

The prevalence of canine dirofilariasis has decreased gradually in China in the past 100 years, but the range of infections has expanded. Before 2000, dirofilariasis was mainly found in southwestern China (Sichuan, Chongqing, and Guizhou) and eastern China (Fujian and Taiwan). Only a few scattered and sporadic studies on dirofilariasis have been carried out in central, southern and northeastern China. After 2000, canine dirofilariasis remained endemic in southern, southwestern and eastern China. The disease was also found in northwestern China, where it had not been reported before 2000. *D. immitis* was also found, primarily in areas in southwestern, southern, central, eastern and northeastern China, where previously only sporadic or negligible numbers of cases had been reported.

The epidemiologic data of the studies included in this review show a change in the distribution of dirofilariasis in China, which has shifted toward northwest, north and eastern China. Many factors might be responsible for this, but some in particular are thought to possibly explain the changes in the occurrence and distribution of canine dirofilariasis in China. The increasing number of dogs traveling with their holidaying owners, or dogs sold in endemic areas and brought to non-endemic areas could be key factors in the spread of infections into new areas. Taking Taiwan as an example, canine dirofilariasis was first reported there, by Miyamoto [36], when one dog imported from Mainland China and another from Japan were found to be infected, while negative results were obtained for 75 indigenous dogs. Since then, a large number of dogs have been imported from areas endemic for dirofilariasis, including the USA and Japan. Wu et al. [66] observed that, throughout Taiwan, a total of 837 stray dogs and 1228 pet dogs were infected with heartworm, and the overall heartworm-positive rate for stray dogs and pet dogs was 57% and 26.5%, respectively.

Another fundamental factor is the presence of mosquitoes that are able to act as vectors of *Dirofilaria*. The geographical extent of dirofilariasis is directly related to that of susceptible mosquito populations. In China, heartworm vectors include *Anopheles sinensis*, *Aedes albopictus* and *Culex pipiens pallens*, and other species of mosquitoes [44]. Among vectors reported worldwide, *An. sinensis* is distributed throughout almost all of China, except for Xinjiang and Qinghai [50], and *Ae. albopictus* is found in 28 provinces/autonomous regions/municipalities [67]. Furthermore, the new introduction into a given area of a competent mosquito species, e.g. *Ae. albopictus*, may also affect dirofilariasis transmission. Previously, *Ae. albopictus* mainly resided south of 30° N in China, but now has a wider distribution in the country, and particularly in Liaoning, Hebei, Shanxi, Shannxi, and Tibet, where it has spread in recent years [19, 48, 67]. Its capacity to transmit infective L3 of *D. immitis* has been confirmed in Fujian province and some other areas of China [31].

Environmental conditions are other important factors affecting mosquito development and the distribution of dirofilariasis. The pivotal prerequisite for *D. immitis* transmission is a climate with a suitable temperature and humidity to support a viable mosquito population, and also maintain sufficient heat to allow the maturation of ingested microfilariae to infective L3 in the mosquitoes. Development and maturation in mosquitoes require a steady temperature in excess of 18 °C for approximately 1 month. Some new areas are becoming more suitable for vectors due to global warming and climatic change, which result in their wider geographical distribution. Large populations of mosquitoes that are able to transmit dirofilariasis, and temperatures that favor the mosquitoes and the development of infectious L3 in them, increase the risk of transmission of the disease [14] and enable the spread of vectors to new areas [39]. Our meta-regression showed a relationship between latitude and *Dirofilaria* infection in dogs which may be related to factors such as climatic conditions or differences in the nutritional and health management of the animals. Dogs in the highlands of western China are used for herding, while dogs on the plains of northern China are used for hunting, and their living conditions and care are worse than those of guard dogs and pet dogs in central and southern China.

Current research on risk factors related to canine dirofilariasis is mainly focused on factors related to the parasites and their vectors. High-quality surveillance data on mosquitoes are notoriously difficult to acquire. Vegetation indices and meteorological data are used as surrogates when mapping the abundances of these mosquito vector species. As the ambient air temperatures experienced by the mosquito vectors affect heartworm transmission, these can be used to predict dog and heartworm vector competence. As the relationships between environmental conditions and *Dirofilaria* infection are the main focus of current research, more attention needs to be paid to host factors. In the present review, age and care of dogs were found to be important factors affecting the infection rate of heartworm. Older dogs had a higher positive rate and were more likely to be bitten by mosquitoes carrying infective L3. This may also explain why stray dogs were more likely to be infected by heartworm. Thus, epidemiological monitoring of domestic dogs and wild canids, in addition to the care and management of dogs, is considered very important for the prevention of canine dirofilariasis.

Although *D. immitis* is most commonly found in dogs in China, the level of *D. immitis* infection in cats should also be taken into consideration. A study reported *D. immitis* prevalence of 3.0% in cats in Gansu, northwestern China [6], Kang et al. [24] reported 1.9% prevalence in stray cats from Liaoning, Jilin, Heilongjiang, and Shandong, and another study reported an average prevalence of *D. immitis* of 4.5% in cats in Liaoning [17]. The overall prevalence of antibody-positive cats was found to be 6.7% in Taiwan in 2017 [33]. *D. immitis* can also infect other mammals, such as foxes, red pandas, leopards, white-lipped deer, giant pandas and hog deer [4, 74, 75].

Although humans are not natural hosts of *Dirofilaria* spp., a few human cases of *Dirofilaria* infection have been reported [3], mostly in the USA and Europe. Human cases have also been reported in China, most of which were attributed to *D. repens*, although other *Dirofilaria* spp. may also infect and cause disease in humans. In 1980, Huang et al. [22] reported two human cases in Heilongjiang province, China, where *D. repens* had infected the eyes. In 1986, Zhang [70] reported one human case of *D. repens* infection in subcutaneous tissue. In 1987, Sun [53] reported one human case of an eye infected with *D. repens* in Heilongjiang province. Two cases of *D. repens* infection in the breast in adult females residing in Hong Kong were reported in 2002 [41]. A case of dirofilariasis affecting the buccal mucosa was reported in a non-endemic area of southern China [56]. Two cases of human pulmonary dirofilariasis have been reported in Taiwan [58]. In 2005, Wang and Cui [64] reported two cases of *D. immitis* infection in humans. Huang et al. [21] reported one human case of subcutaneous dirofilariasis caused by *D. immitis* in Taiwan. To et al. [57] reported the detection of a novel species, “*Candidatus* Dirofilaria hongkongensis,” which was responsible for three cases of human dirofilariasis in Hong Kong. Cheung et al. [5] reported that a patient was diagnosed with *Dirofilaria* infection from a subcutaneous nodule on the right thigh. In 2013, Li [29] reported one case of human pulmonary dirofilariasis concurrent with intercostal neurilemmoma in Taiwan. Considering the risk that *D. repens* poses to human health, an investigation of *D. repens* infection among dogs needs to be started immediately in China.

The limitations of this systematic review include the following: (1) only a few of the included studies had evaluated the multiple factors that influence dirofilariasis, (2) there was insufficient data from the studies for subgroup analysis, (3) epidemiological survey results were missing for some provinces and cities of China, and (4) the data were highly heterogeneous.

## Conclusions

The pooled prevalence of dirofilariasis in dogs in China as determined in the meta-analysis presented here shows that the disease is widespread in the country. In the past 100 years, the prevalence of canine dirofilariasis in previously endemic and hyper-endemic areas of China has slowly decreased, but infections have spread into previously infection-free areas of the country. In addition to factors associated with the parasites and their vectors, attention should also be paid to factors that affect the canine hosts, as should the epidemiological monitoring and management of dogs. More attention should also be paid to increasing the public’s awareness of the zoonotic nature of dirofilariasis. Moreover, veterinarians need to develop more effective detection methods for the disease and take appropriate measures to prevent its spread and manage its treatment.

## Supplementary Information

**Additional file 1: Figure S1.** Publication bias and sensitivity analysis for the meta-analysis.** A** Funnel plot for the examination of publication bias,** B** Egger’s test for publication bias,** C** Funnel plot with trim-and-fill analysis of publication bias,** D** Sensitivity analysis of any one included study.

**Additional file 2: Table S1.** Included studies on prevalence of canine dirofilariasis in China.

**Additional file 3: Table S2.** Eligible data included in the study.

**Additional file 4: **Preferred reporting items for systematic reviews and meta-analyses checklist items.

## Data Availability

The datasets used during the study are available from the corresponding author upon reasonable request.
