# Supplementary material for: The prevalence of canine dirofilariasis in China: a systematic review and meta-analysis
Source: Parasit Vectors. 2023 Jun 20;16:207. doi: 10.1186/s13071-023-05770-9 (PMC10283191; doi:10.1186/s13071-023-05770-9)
Supplement: Supplementary file 2 — Additional file 2: Table S1. Included studies on prevalence of canine dirofilariasis in China. [file 13071_2023_5770_MOESM2_ESM.docx]

**Table S1****.** Included studies on prevalence of canine dirofilariasis in China

| **Study** | **No. tested** | **No. positive** | **Prevalence** | **Score** | **References** |
| --- | --- | --- | --- | --- | --- |
| Chen et al., 1992 | 122 | 15 | 12.30% | 4 | [76] |
| Chen et al., 1996 | 106 | 11 | 10.38% | 5 | [78] |
| Chen et al., 2012 | 4787 | 19 | 0.40% | 7 | [77] |
| Chou et al., 2014 | 720 | 52 | 7.22% | 7 | [79] |
| Dong et al., 2004 | 178 | 49 | 27.53% | 3 | [80] |
| Fan et al., 2001 | 664 | 89 | 13.40% | 7 | [81] |
| He et al., 2005 | 16 | 5 | 31.25% | 6 | [83] |
| He et al., 2010 | 300 | 15 | 5.00% | 1 | [82] |
| Hou et al., 2006 | 1048 | 237 | 22.61% | 7 | [84] |
| Hou et al., 2011 | 886 | 213 | 24.04% | 7 | [111] |
| Lin et al., 1986 | 808 | 1 | 0.12% | 4 | [85] |
| Liu et al., 1995 | 15 | 5 | 33.33% | 4 | [88] |
| Liu et al., 2005 | 95 | 2 | 2.11% | 2 | [87] |
| Liu et al., 2013 | 528 | 67 | 12.69% | 7 | [86] |
| Lu et al., 2017 | 2064 | 471 | 22.82% | 7 | [33] |
| Qian et al., 1996 | 273 | 40 | 14.65% | 4 | [43] |
| Quan et al., 2016 | 477 | 1 | 0.21% | 7 | [89] |
| Rao et al., 1999 | 31 | 19 | 61.29% | 7 | [90] |
| Shen et al., 1984 | 100 | 4 | 4.00% | 4 | [91] |
| Sun et al., 1987 | 15 | 12 | 80.00% | 4 | [50] |
| Sun et al., 2012 | 310 | 42 | 13.55% | 7 | [51] |
| Wang et al., 1988 | 50 | 0 | 0.00% | 4 | [98] |
| Wang et al., 1995 | 120 | 45 | 37.50% | 4 | [62] |
| Wang et al., 1997 | 180 | 99 | 55.00% | 7 | [95] |
| Wang et al., 2006 | 178 | 2 | 1.12% | 5 | [92] |
| Wang et al., 2012 | 26 | 0 | 0.00% | 2 | [96] |
| Wang et al., 2016 | 1176 | 155 | 13.18% | 7 | [97] |
| Wang et al., 2018 | 1037 | 1 | 0.10% | 6 | [93] |
| Wang et al., 2019 | 1038 | 65 | 6.26% | 6 | [94] |
| Wu et al., 1956 | 400 | 155 | 38.75% | 4 | [99] |
| Wu et al., 2003 | 2125 | 803 | 37.79% | 7 | [66] |
| Xia, et al., 2012 | 600 | 7 | 1.17% | 7 | [100] |
| Xie et al., 1992 | 85 | 37 | 43.53% | 5 | [101] |
| Xu et al., 1995 | 46 | 1 | 2.17% | 7 | [103] |
| Xu et al., 2015 | 1114 | 0 | 0.00% | 7 | [102] |
| Yang et al., 2002 | 50 | 40 | 80.00% | 3 | [104] |
| Ye et al., 2012 | 128 | 26 | 20.31% | 7 | [105] |
| Yuasa et al., 2012 | 344 | 71 | 20.64% | 7 | [106] |
| Zhang et al., 2008 | 28783 | 6 | 0.02% | 2 | [107] |
| Zhang et al., 2010 | 210 | 0 | 0.00% | 7 | [108] |
| Zhou et al., 2013 | 20 | 11 | 55.00% | 4 | [109] |
| Zhu et al., 2017 | 60 | 3 | 5.00% | 7 | [110] |

Note: The references were listed in the Reference section in the main text.
