# Supplementary material for: The prevalence of canine dirofilariasis in China: a systematic review and meta-analysis
Source: Parasit Vectors. 2023 Jun 20;16:207. doi: 10.1186/s13071-023-05770-9 (PMC10283191; doi:10.1186/s13071-023-05770-9)
Supplement: Supplementary file 4 — Additional file 4: Preferred reporting items for systematic reviews and meta-analyses checklist items. [file 13071_2023_5770_MOESM4_ESM.doc]

**PRISMA-P (Preferred Reporting Items for Systematic review and Meta-Analysis Protocols) 2015 checklist: recommended items to address in a systematic review protocol***

| Section and topic | Item No | Checklist item | Reported on page # |
| --- | --- | --- | --- |
| ADMINISTRATIVE INFORMATION | | |  |
| Title: |  |  |  |
| Identification | 1a | The report is identified as a systematic review and meta-analysis. | 1 |
| Update | 1b | The report is not an update of a previous systematic review. |  |
| Registration | 2 | Registration does not apply. |  |
| Authors: |  |  |  |
| Contact | 3a | Contact information is provided in the report. | 1 |
| Contributions | 3b | Described in the report. | 19 |
| Amendments | 4 | The report states plan for documenting important protocol amendments. |  |
| Support: |  |  |  |
| Sources | 5a | This work was funded by the Major Science and Technology Plan of Hainan Province (ZDKJ2021035) and the National Natural Science Foundation of China [U22A20363]. | 18 |
| Sponsor | 5b |  |  |
| Role of sponsor or funder | 5c | No. |  |
| INTRODUCTION | | |  |
| Rationale | 6 | Described in the introduction. | 3-4 |
| Objectives | 7 | Stated in the introduction. | 3-4 |
| METHODS | | |  |
| Eligibility criteria | 8 | Selection criteria is provided in the methods. | 5 |
| Information sources | 9 | All articles were retrieved via major English (PubMed and Web of Science) and Chinese databases (CNKI, VIP, and Wan Fang databases) before December 2022 | 5 |
| Search strategy | 10 | Search strategy is provided in the methods. | 5 |
| Study records: |  |  |  |
| Data management | 11a | Described in the item 2.3. | 5 |
| Selection process | 11b | Stated in the methods. | 5 |
| Data collection process | 11c | Described in the methods. | 5 |
| Data items | 12 | Described in the methods. | 5 |
| Outcomes and prioritization | 13 | Listed in the results. | 6 |
| Risk of bias in individual studies | 14 | Described in the results. | 7 |
| Data synthesis | 15a | Described in the results. | 7 |
|  | 15b | Described in the results. | 7 |
|  | 15c | Described in in the results. | 8 |
|  | 15d | Quantitative synthesis is appropriate in this report. | 8 |
| Meta-bias(es) | 16 | Described in the results. | 8 |
| Confidence in cumulative evidence | 17 | Described in the results. | 8 |

*** It is strongly recommended that this checklist be read in conjunction with the PRISMA-P Explanation and Elaboration (cite when available) for important clarification on the items. Amendments to a review protocol should be tracked and dated. The copyright for PRISMA-P (including checklist) is held by the PRISMA-P Group and is distributed under a Creative Commons Attribution Licence 4.0.**

*From: Shamseer L, Moher D, Clarke M, Ghersi D, Liberati A, Petticrew M, Shekelle P, Stewart L, PRISMA-P Group. Preferred reporting items for systematic review and meta-analysis protocols (PRISMA-P) 2015: elaboration and explanation. BMJ. 2015 Jan 2;349(jan02 1):g7647.*
